# Revising the motivation and confidence domain of the Canadian assessment of physical literacy

**DOI:** 10.1186/s12889-018-5900-0

**Published:** 2018-10-02

**Authors:** Katie E. Gunnell, Patricia E. Longmuir, Sarah J. Woodruff, Joel D. Barnes, Kevin Belanger, Mark S. Tremblay

**Affiliations:** 1 Department of Psychology, Carleton University, A511 Loeb Building, 1125 Colonel By Drive, Ottawa, ON K1S 5B6 Canada; 2 Healthy Active Living and Obesity (HALO) Research Group, Children’s Hospital of Eastern Ontario Research Institute, Ottawa, K1H 8L1 Canada; 3 Department of Paediatrics, Faculty of Medicine, University of Ottawa, Ottawa, K1N 6N5 Canada; 4 Department of Kinesiology, University of Windsor, Windsor, N9B 3P4 Canada

**Keywords:** Intrinsic motivation, Physical activity, Competence, Children, Adequacy, Predilection

## Abstract

**Background:**

The Motivation and Confidence domain questionnaire in the Canadian Assessment of Physical Literacy (CAPL) was lengthy (36 single items that aggregate to five subscales), and thus burdensome to both participants and practitioners. The purpose of this study was to use factor analysis to refine the Motivation and Confidence domain to be used in the CAPL–Second Edition (CAPL-2).

**Methods:**

Children, primarily recruited through free-of-charge summer day camps (*n* = 205, M_age_ = 9.50 years, SD = 1.14, 50.7% girls), completed the CAPL-2 protocol, and two survey versions of the Motivation and Confidence questionnaire. Survey 1 contained the Motivation and Confidence questionnaire items from the original CAPL, whereas Survey 2 contained a battery of items informed by self-determination theory to assess motivation and confidence. First, factor analyses were performed on individual questionnaires to examine validity evidence (i.e., internal structure) and score reliability (i.e., coefficient H and omega total). Second, factor analyses were performed on different combinations of questionnaires to establish the least burdensome yet well-fitted and theoretically aligned model.

**Results:**

The assessment of adequacy and predilection, based on 16 single items as originally conceptualized within the CAPL, was not a good fit to the data. Therefore, a revised and shorter version of these scales was proposed, based on exploratory factor analysis. The self-determination theory items provided a good fit to the data; however, identified, introjected, and external regulation had low score reliability. Overall, a model comprising three single items for each of the following subscales was proposed for use within the CAPL-2: adequacy, predilection, intrinsic motivation, and perceived competence satisfaction. This revised domain fit well within the overall CAPL-2 model specifying a higher-order physical literacy factor (MLRχ^2^_(63)_ = 81.45, *p* = 0.06, CFI = 0.908, RMSEA = 0.038, 90% CI (0.00, 0.060)).

**Conclusions:**

The revised and much shorter questionnaire of 12 items that aggregate to four subscales within the domain of Motivation and Confidence is recommended for use in the CAPL-2. The revised domain is aligned with the definition of motivation and confidence within physical literacy and has clearer instructions for completion.

**Electronic supplementary material:**

The online version of this article (10.1186/s12889-018-5900-0) contains supplementary material, which is available to authorized users.

## Background

The Canadian Assessment of Physical Literacy (CAPL) [1] is a comprehensive instrument designed to assess children’s physical literacy. Physical literacy can be defined as “the motivation, confidence, physical competence, knowledge and understanding to value and take responsibility for engagement in physical activities for life” [2]. Although debate exists as to whether or not physical literacy needs to be and can be quantified [3], some have argued that appropriate measurement of physical literacy should be established if physical literacy is to serve as a key outcome of physical education curricula [4]. The CAPL comprises four interrelated domains: Physical Competence; Daily Behaviour; Motivation and Confidence; and Knowledge and Understanding.

Recently, confirmatory factor analyses were used to refine the 25 aggregated indicators of the CAPL, and results supported the factor structure of a 14-aggregated-indicator version called the CAPL–Second Edition (CAPL-2; see [5]). Nevertheless, this move toward a more parsimonious model did not successfully resolve issues about participant and administrator burden or about the theoretical anchoring for the Motivation and Confidence domain. Therefore, the overall objectives of this paper were to: (1) further refine the CAPL Motivation and Confidence domain by reducing the number of items participants needed to complete and by enhancing instructional clarity; and (2) ensure the Motivation and Confidence domain was theoretically aligned with the definitions of motivation and confidence within the context of physical literacy and a theory of motivation.

### Motivation and confidence in CAPL: Current operationalization and issues

In an effort to operationalize Motivation and Confidence within the CAPL, a variety of self-reported motivational items were assembled. First, Whitehead’s writing on physical literacy [6], particularly on motivation and confidence, was reviewed. Second, pre-existing questionnaires with evidence of score reliability and validity in children were selected from among those that assessed constructs related to the concept of motivation and confidence in physical literacy as described by Whitehead. An advisory panel of scientists, educators, and policy makers with expertise in childhood physical activity reviewed the proposed measures, and those measures that were most strongly supported were retained. The final assessments included: (1) a questionnaire assessing the self-perceived benefits (9 items) and barriers (10 items) to physical activity, which was then used to create a benefits-to-barriers difference score [7]; (2) two questionnaire subscales assessing children’s self-perceptions of adequacy (7 items) and predilection (9 items) for physical activity [8]; one item assessing perceived risk of injury during physical activity that was not used within CAPL calculated scores; and (3) two items that assessed children’s perceived activity level and skill compared to others on a 10-point Likert scale. Once the questionnaires were implemented, feedback from researchers, teachers, and coaches who had used the CAPL was used to identify problematic areas within the Motivation and Confidence domain, specifically around participant burden (i.e., the length of time required to complete the combined scales), and instructional clarity.

In reporting the results of an international Delphi panel validation of the CAPL, Francis and colleagues [9] called for further refinement and validation work on the Motivation and Confidence domain of CAPL. Concerns raised during the CAPL Delphi process (see round 2b) suggested the experts did not agree that all of the items selected to assess motivation and confidence should be included [9]. Consequently, we re-evaluated the measures used in CAPL to assess motivation and confidence alongside theory and definitions forwarded within the physical literacy framework. Within the framework of physical literacy, Whitehead [6] defined motivation as the “desire to be active, to persist with an activity, to improve physical competence and to try new activities” (p. 30). She hypothesized that people who had greater physical literacy would also “be confident in their physical abilities knowing that success is likely” (p. 30).

After carefully inspecting the original questionnaires within the Motivation and Confidence domain in comparison to Whitehead’s definition presented above, we recognized that some aggregated items may be better characterized as more distal indicators of motivation and confidence rather than proximal indicators. Furthermore, we noted that Whitehead’s definition focused on the positive aspects of motivation and confidence for physical activity, rather than the negative aspects or factors that detracted from motivation and confidence. In other words, the definition focused on why people with higher physical literacy engage in physical activity, and not why those with lower physical literacy avoid physical activity. Consequently, we contend that perceived benefits (which represent a cognitive appraisal of the benefits associated with being physically active) may be a distal indicator of motivation and confidence. Similarly, perceived barriers (which represent hindrances or blocks to physical activity [7]) might not align with the types of motivation and confidence denoted within some physical literacy literature.

In re-evaluating the conceptualizations of motivation and confidence in the original CAPL, it became apparent that the domain was not sufficiently grounded in a specific theory of motivation that had empirical evidence within physical activity contexts. Whitehead’s description of motivation and confidence is somewhat vague, and does not map directly onto specific definitions of motivation and confidence outlined within theories of motivation (or similar constructs such as self-efficacy or competence). Nonetheless, theoretically anchoring the definition of motivation and confidence within the CAPL is critical to the advancement of measuring this important domain of physical literary. Indeed, using a theoretical framework of motivation can both complement Whitehead’s definition of motivation and confidence and extend it by enhancing precision of measurement and predictive abilities.

One theory that is often used to understand the quality of motivation and perceived competence is self-determination theory [10], which was also reviewed as relevant to the understanding of motivation within the context of physical literacy [11]. According to this theory, motivation for physical activity can be regulated along a continuum based on the degree to which the behaviour is autonomous [10]. The most extrinsic form of motivation is extrinsic regulation (e.g., feeling pressured to engage in physical activity by an external force). Underpinned by increasing autonomy, other forms of extrinsic motivation are introjected regulation (e.g., feeling pressured by internal forces such as guilt, shame, or pride); identified regulation (e.g., identifying the value and benefits of the behaviour); and integrated (e.g., engaging in the behaviour because it represents a part of one’s identity). The most autonomous form of motivation is intrinsic motivation (e.g., engaging in an activity because it is enjoyable and fun [10]). Self-determination theory also highlights a fundamental psychological need to experience competence (e.g., feeling effective in ongoing interactions within an environment [10]). Intrinsic motivation and perceived competence are complementary to Whitehead’s description of motivation and confidence [6]. Further, adding motivational regulation of physical activity and perceived competence to the Motivation and Confidence domain of CAPL extends the precision of measurement and predictive validity. Consequently, because of our re-evaluation of the theoretical alignment of the Motivation and Confidence domain, we hypothesized that the self-determination theory constructs of motivation and perceptions of competence could be added alongside existing items to theoretically anchor this CAPL domain.

Another issue related to the CAPL Motivation and Confidence domain pertained to participant burden and instructional clarity. CAPL administrators (i.e., people who administer the CAPL to children, such as researchers, coaches, and teachers) had informally reported to the study coordinating centre that, if children independently read the instructions and practice questions rather than reviewing them orally as a group, then some children had trouble understanding how to answer. Within the CAPL, the items assessing perceived adequacy (i.e., self-perception that one has the capability to achieve an acceptable standard of success contextualized by perceptions of the self, parents, teachers, peers, and societal expectations), perceived predilection (i.e., the likelihood that an individual will select physical activity over sedentary behaviour when given the choice), and perceived injury-risk, all used a structured alternative response format [8]. With structured alternative response formats, items contain two contrasting statements, and participants are first asked to pick which one is “more like them” and, second, to indicate “how true that item is for them”. It is believed that this well-established response format reduces social desirability responding, and is more readily understood by younger children. When the instructions are given with care by an administrator, the format can be appropriate [12]. However, there have also been reports that the structured response format creates statistical method effects (i.e., covariation that comes from factors unrelated to the variables of interest [13, 14]) and can be confusing for children who are not properly instructed in how to respond [15]. We therefore wanted to examine whether enhancing the clarity of the instructions for the structured alternative format items, and ensuring that the administrators were careful to clearly explain how to respond to the items, would improve clarity for the participants. Second, we wanted to examine responses with statistical analyses to verify the factor structure of the scores.

Lastly, CAPL administrators had identified comprehension issues pertaining to the perceived barriers items that were used as one component of the benefits-to-barriers difference score [7]. These items were presented in a Likert response format. In particular, they contained double negatives that have been shown to be confusing to children [16]. For example, the instructional stem has children read, “I might not be active if…” and the possible answers to that stem also contain negative wording (e.g., “I didn’t have enough time to be active”). Based on this informal feedback, and the lack of theoretical alignment identified above, we wanted to use statistical modelling to re-evaluate the Motivation and Confidence domain without the perceived benefits-to-barriers difference score.

### Purpose and hypotheses

The purpose of this study was to explore refinements to the CAPL Motivation and Confidence domain to address the issues identified above. Specifically, we sought to: (1) reduce the number of items participants needed to complete; (2) enhance instruction clarity; (3) ensure that the items within the Motivation and Confidence domain were more closely aligned with well-supported motivational theory; and (4) ensure that these items demonstrated good factor structure and reliability. It is important to note that our purpose was to use existing questionnaires that have demonstrated initial score reliability and validity in children and youth for the assessment of motivation and confidence. Our intention was not to re-develop items, item response formats, or create new items. Rather, our goal was to refine existing CAPL questionnaires and add existing questionnaires to theoretically anchor the Motivation and Confidence domain within CAPL. We view the development of CAPL as an ongoing process, and this contribution should be seen as one initial step in the ongoing process of validation.

Finally, although we recognize that Whitehead’s concept of charting progress in physical literacy is well aligned to objective measurements [6], the CAPL was specifically developed to address calls for the development of standardized assessments of physical literacy [4]. To this end, the purpose of this contribution was not to advance the debate about how or whether physical literacy should (or should not be) measured; rather, it was to refine the motivation and confidence component of one standardized assessment of physical literacy.

## Methods

Children (*n* = 205, M_age_ = 9.50 years, SD = 1.14 years, 50.7% girls) who were enrolled in YMCA free summer camps in southwestern Ontario completed the CAPL-2 (see [5] plus the revised surveys in Additional files 1 and 2). The revised surveys were the original CAPL measures of motivation (Survey 1), and the self-determination theory-based measures of motivation plus the revised CAPL Knowledge and Understanding questionnaire (Survey 2) (see [17]). The CAPL-2 was administered by a research assistant who was trained to administer the CAPL. This study received ethical approval through the research ethics boards of both the Children’s Hospital of Eastern Ontario Research Institute and the University of Windsor. Parents were asked to complete written informed consent when they dropped their child off at the camp, and children provided verbal assent on the day of testing. Children were provided with a description of the study, including what participation entailed, and were reminded that they did not have to do anything they did not want to do. The total number of participants approached to participate was not collected. In total, 233 participants provided informed consent/assent. Of these, 11 participants refused to participate in the assessments and/or were absent on the day of data collection. An additional 17 were removed due to violations of age/gender/interquartile range rules (see data analysis section below).

In all cases, participants were split into two groups; each group first completed either the physical testing or the surveys (i.e., Survey 1 or Survey 2 delivered in a randomized order for each YMCA camp) and then alternated to the other activity. Upon completion, each participant was then given a pedometer with instructions on how it should be worn and how to use the recording sheet.

### Measures

#### CAPL motivation and confidence questionnaire (survey 1; see Additional file 1)

The original Motivation and Confidence domain of CAPL–First Edition (CAPL-1) contained five subscales. Adequacy and predilection for physical activity were assessed using those subscales from the Children’s Self-perceived Adequacy and Predilection for Physical Activity Scale questionnaire [8]. Although the original scale and CAPL-1 questionnaires contained one item assessing injury, only adequacy and predilection subscales were used within CAPL [9]. The adequacy and predilection items were presented using a structured alternative response format. For example, children first read one item (e.g., “Some kids can’t wait to play active games after school BUT other kids would rather do something else after school”) and were then asked to circle which of the two statements was most like them. Then children were asked to indicate if their circled response is “really true for me” or “sort of true for me”.

Likelihood to pursue physical activity was assessed with a benefits-to-barriers difference score, derived from the self-perceived benefits and barriers questionnaire [7]. Children read 10 barriers items (e.g., following the stem “I might not be active if…”, an example item was “I didn’t have enough time to be active”) and nine benefits items (e.g., following the stem “A reason that I might be active is because when I am active…”, an example item was “…I look better”). The children were asked to rate each item on a scale of 1 (*disagree a lot*) to 5 (*agree a lot*). The benefits-to-barriers difference score was created by subtracting the total barrier score from the total benefits score.

Finally, the final concept (assessed with one item) asked children, “Compared to other kids your age, how good are you at sports or skills?”; children rated their responses on a scale of 1 (*others are better*) to 10 (*I’m a lot better*). There was one additional concept assessed with one item in the original CAPL (i.e., “Compared to other kids your age, how active are you?”), but it was not assessed in this study, based on previous findings (see [5]).

#### Proposed self-determination theory-based motivation and confidence questionnaire (survey 2; see Additional file 2)

The 12 items from the child-adapted version of the Behavioural Regulation in Exercise Questionnaire [18, 19] were used to assess motivational regulation for physical activity. Using three single items each, the questionnaire assessed: intrinsic motivation (i.e., pursuing activity for pleasure and fun; “being active is fun”); identified regulation (i.e., pursuing activity because you value the benefits; “it is important to me to be active”); introjected regulation (i.e., pursuing activity because you feel guilt or shame if you do not; “when I’m not active I feel bad”); and external regulation (i.e., pursuing activity because of external pressure such as through a parent; “other people say I should be”). Children read each statement and responded on a Likert scale ranging from 1 (*not true for me*) to 5 (*very true for me*). Additionally, children completed the six-item subscale from a child-adapted version [19] of a scale that had been previously developed to assess competence for physical activity [20]. Children were asked to read each item (e.g., “When it comes to playing active games, I think I am pretty good”) and to then respond on a Likert scale ranging from 1 (*not like me at all*) to 5 (*really like me*). Both instruments were adapted for children, and aligned with theoretical tenets outlined within self-determination theory [19]. In our study, after the first 33 children completed the items, we made a slight modification to enhance item clarity. Specifically, we contextualized three items from the extrinsic regulation subscale (e.g., the original item read: “Other people say I should be…” and the modified item read: “Other people say I should be active”), and one item for competence, to situate the items during “activity” (see Additional file 3, Tables 1 and 2, for wording modifications).

**Table 1:** Model fit statistics for different questionnaires to assess motivation and confidence constructs

	Chi-square	Df	*P*	CFI	RMSEA	RMSEA 90% CI	λ range
Adequacy and predilection (original)	291.99	103	< 0.001	0.744	0.095	0.082, 0.107	0.301–0.698
Adequacy and predilection (revised)	29.21	32	0.609	1.0	0.00	0.000, 0.046	0.531–0.836
Benefits and barriers (original)	230.06	151	< 0.001	0.894	0.051	0.037, 0.063	0.359–0.768
Benefits and barriers (revised)	189.20	150	0.017	0.948	0.036	0.016, 0.051	0.349–0.783
Perceived competence (original)	6.87	9	0.650	1.0	0.000	0.000, 0.064	0.115–0.765
Perceived competence (revised)	2.94	5	0.710	1.0	0.000	0.000, 0.072	0.475–0.767
Motivation	92.16	48	< 0.001	0.907	0.067	0.046, 0.087	0.427–0.859

*Note.* Adequacy and predilection original = original CAPL model with 16 items. Adequacy and predilection (revised) = reduced model based on EFA results with 10 items and 3 subscales representing adequacy, predilection, and behaviour. Benefits and barriers (original) = original model with 19 items. Benefits and barriers (revised) = revised model with a correlated error. Perceived competence (original) = perceived competence with 6 items. Perceived competence (revised) = perceived competence excluding the reverse coded item. Motivation = intrinsic, identified, introjected, and extrinsic regulation subscales

*CFI* comparative fit index, *CI* 90% confidence interval, *Df* degrees of freedom, *EFA* exploratory factor analysis, *RMSEA* root mean square error of approximation, *λ* standardized factor loading

**Table 2:** Model fit statistics and reliability for different models of Motivation and Confidence

	Chi-square	Df	*P*	CFI	RMSEA	RMSEA 90% CI	λ range	Total # of items
Model 1	20.64	2	<.0001	0.877	0.213	0.136, 0.301	0.482–0.796	36
Model 2	16.62	5	0.005	0.946	0.106	0.053, 0.165	0.219–0.741	18
Model 3	13.80	5	0.02	0.923	0.093	0.036, 0.153	0.439–0.663	14
Model 4	4.24	2	0.12	0.978	0.074	0.000, 0.174	0.490–0.760	12

*Note.* Model 1 = composite scores of original adequacy, original predilection, benefits, barriers, and skills compared to peers. Model 2 = composite scores of intrinsic, identified, introjected, and external regulation as well as competence. Model 3 = composite scores of intrinsic regulation, skill compared to peers, shortened adequacy and shortened predilection, and a behaviour subscale. Model 4 = composite scores of 3 items each for adequacy, predilection, competence, intrinsic motivation

*CFI* comparative fit index, *CI* 90% confidence interval *Df* degrees of freedom, *RMSEA* root mean square error of approximation, *λ* standardized factor loading

In total, 33 children completed the originally worded items and 172 children completed the modified items. Given how slight the wording modifications were, analyses presented herein included all children; however, a sensitivity analysis was run on our final model removing the 33 children who completed the original items, to confirm that the results did not change based on the slight wording modification.

#### Knowledge and understanding

This domain was assessed with five items [5]. Compared to the previous versions of CAPL (see [5]), the items used in this survey to assess knowledge of physical activity guidelines had altered response options [21]. Children were asked, “How many minutes each day should you and other children do physical activities that make your heart beat faster and make you breathe faster, like walking fast or running? Count the time you should be active at school and also when you are at home or in your neighborhood”. Response options included: “20 min”; “30 min”; “60 minutes or 1 hour”; and “120 minutes or 2 hours” – with the correct response being “60 min”. The score for this item was 0 (incorrect) or 1 (correct). Additionally, the comprehension subscale contained one additional fill-in-the-blank answer, and was scored out of 7 for each correct word in the appropriate blank space [21].

#### Daily behaviour

This domain was measured via self-report questionnaire and pedometer step counts (see [5]). Children were asked to report the number of days they engaged in moderate or vigorous physical activity in a typical week, ranging from 0 (*days*) to 7 (*days*). An SC-StepRx pedometer (StepsCount, Deep River, ON) was worn over the right hip to assess how many steps were taken each day over a week [22]. A score was considered valid if the child wore the pedometer for at least 10 h per day on at least 4 days in the week, with step counts between 1000 and 30,000 steps per day.

#### Physical competence

This domain was assessed with three composite indicators. First, children completed the isometric plank without a time limit [23] and scores were recorded to the nearest second. Next, children completed the Canadian Agility and Movement Skill Assessment [24], and their performance and time taken to complete the skills was recorded. Lastly, the Progressive Aerobic Cardiovascular Endurance Run ([25] was completed and scored in number of laps completed.

### Data analyses

Data screening and cleaning was conducted in R using the psych package [26, 27]. Participants were removed (*n* = 17; [28]) if they did not provide data on age or gender, or if their scores fell outside 1.5*Interquartile range [28]. Age- and gender-matched z-scores were calculated for each variable, and no outliers (z > 5.00) were present. Descriptive statistics for each item are presented in Additional file 4. The main analyses were estimated in Mplus version 8.0. All syntax is provided in Additional file 5.

Analyses proceeded in sequential steps. In the first step, confirmatory factor analyses were calculated separately for each individual measurement scale. Coefficient H and omega total were calculated as estimates of score reliability for each subscale (formulas provided in Additional file 5). Coefficient H is an assessment of maximal reliability based on factor loadings derived from the factor analysis, assuming optimal weighting (i.e., every item contributes different amounts to the total scale score) [29]. Omega total is an assessment of reliability based on factor loadings and error variances that assume unit-weighting (i.e., every item contributes equally to the total scale score) [30]. Both indicators of reliability are presented to inform readers because they are superior to alpha, assume congeneric models, and provide different information depending on the goal of the researcher. For example, coefficient H will provide an estimate of score reliability assuming a researcher is using optimal weighting (e.g., through factor analysis), whereas omega total will provide an estimate of reliability assuming the researcher is adding up raw items to create a total scale score (e.g., using manifest variable models such as regression).

In the second step, confirmatory factor analyses specifying measurement models, which comprised various combinations of motivation and confidence based on composite scores, were specified and evaluated. Composite scores were used in this step given the complexity of the overall CAPL models and the small sample size. Two models hypothesized a priori were tested. The first was the original CAPL-1 model excluding activity compared to others (i.e., composite scores of adequacy, predilection, benefits-to-barriers difference, and skill compared to others; Survey 1). The second was the self-determination theory-based measures (i.e., composite scores of intrinsic, identified, introjected, and external regulation as well as perceived competence satisfaction; Survey 2). Other exploratory models were informed by the results of the individual confirmatory factor analysis in Step 1, and comprised of a mix of questionnaires from Survey 1 and Survey 2.

In the third step, the final selected model from Step 2 was entered into a measurement model with all other CAPL-2 domains, to determine if the revised Motivation and Confidence domain demonstrated a good fit with the other CAPL-2 domains.

In all analyses, the latent factors were identified through constraining their variance to one, and freeing the first item. Robust maximum likelihood (MLR) was used to estimate all Motivation and Confidence models. Mean- and variance-adjusted weighted least square (WLSMV) was used in the final model, with all CAPL-2 domains included given the categorical nature of the Knowledge and Understanding items. A combination of indices was used to interpret model fit [13]. MLRχ^2^ values, which compare the data-model fit (*p* > 0.05 suggests a good fit), were provided but not interpreted given their sensitivity to sample size [13]. A comparative fit index (CFI) close to or above 0.90, and a root mean square error of approximation (RMSEA) value close to or below 0.08, were used as indicators of a good fit [13, 31]. Additionally, all parameter estimates were interpreted to ensure that there were no out-of-range values (e.g., standardized values above 1, negative residual variance) and to inspect the magnitude of the parameter estimates.

## Results

### Step 1: Individual confirmatory factor analyses

#### CAPL motivation and confidence questionnaire

Scores from the adequacy and predilection subscales did not provide a good fit to the data (see Table 1). Modification indices suggested several correlated errors and two cross-loadings from predilection to two items of adequacy, which could indicate the presence of method effects. An exploratory factor analysis was estimated to determine if there was a different factor structure for these items. Results of the exploratory factor analysis with MLR suggested that a four-factor model fit the data best (MLRχ^2^_(62)_ = 80.12, *p* = 0.06, CFI = 0.982, RMSEA = 0.038, 90% CI [0.000, 0.060]). Interpretation of the geomin rotated loadings indicated that four items loaded strongly (range = 0.51–0.85, *p*s <  0.05) onto a factor that matched “adequacy”; three items loaded strongly (range = 0.58–0.68) onto a factor that matched “predilection”; and three items loaded (range = 0.37–0.82, *p*s <  0.054) onto a factor we labelled “behaviour” because the items reflected activities in which the children actually engaged. The fourth factor had a mix of weak and strong factor loadings (range = 0.33–0.85, *p*s < 0.05) that did not have an apparent pattern. The fourth factor comprised a mix of negatively worded predilection and adequacy items. We therefore re-estimated a confirmatory factor analysis using the first three latent factors and omitting the items that loaded onto the fourth latent factor, and the resultant data were a good fit to the model (see Table 1). Score reliability was good for the “behaviour” (Coefficient H = 0.76, omega total = 0.71), the shortened adequacy (Coefficient H = 0.80, omega total = 0.79) and the shortened predilection (Coefficient H = 0.83, omega total = 0.80) subscales. This model was retained for further analysis.

Next, a separate confirmatory factor analysis was estimated specifying perceived benefits as one subscale and perceived barriers as a second correlated subscale; results indicated that the model could be improved (see Table 1). Modification indices suggested adding one error covariance between two benefit items, and this improved model fit (see Table 1). Score reliability of the benefits (Coefficient H = 0.78, omega total = 0.83) and barriers (Coefficient H = 0.76, omega total = 0.78) subscales were good. A confirmatory factor analysis could not be estimated for the single item representing skill compared to others.

#### Proposed self-determination theory motivation and confidence questionnaire

In a confirmatory factor analysis, the perceived competence satisfaction subscale provided a good fit to the data (see Table 1); however, the factor loading on the reverse scored item was low (λ = 0.12, *p* = 0.19). Consistent with Sebire and colleagues [19], we removed the reverse worded item, and the model provided a good fit (see Table 1). Coefficient H was 0.81 and omega total was 0.83 for the competence subscale. In a separate confirmatory factor analysis, the motivation items adapted by Sebire et al. [19] provided a good fit to the data (see Table 1). Score reliability was good for intrinsic motivation (Coefficient H = 0.84, omega total = 0.82), but relatively weak or poor for identified (Coefficient H = 0.66, omega total = 0.65), introjected (Coefficient H = 0.58, omega total = 0.57), and extrinsic (Coefficient H = 0.51, omega total = 0.50) regulations.

### Step 2: A revised motivation and confidence domain

Having established good factor structures for each of the separate questionnaire scores above, we tested a series of models to determine what combination of measures would provide the best physical literacy Motivation and Confidence assessment. All of these models used composite scores rather than individual items as the subscale (e.g., the average of all adequacy items was obtained to serve as the adequacy composite score). Model 1 comprised the original CAPL model (i.e., composite scores of original adequacy, original predilection, benefits-to-barriers difference score, and skills compared to peers; Survey 1). Model 2 comprised only the self-determination theory based measures (i.e., composite scores of intrinsic, identified, introjected, and external regulation as well as perceived competence satisfaction; Survey 2). The subsequent two models were exploratory and based on results from the individual confirmatory factor analyses obtained in Step 1 as well as theory. Model 3 comprised a select subset of measures from both the original CAPL (Survey 1) and the self-determination theory instruments (Survey 2). Model 3 was made up of composite scores of intrinsic regulation, skill compared to peers, shortened adequacy and shortened predilection, and behaviour subscale.

Model 1, the original CAPL model (Survey 1) provided a poor fit to the data (see Table 2). Model 2, the self-determination theory-based measures (Survey 2) provided a good fit to the data (see Table 2); however, the extrinsic regulation subscale had a weak factor loading (λ = 0.22, *p* = 0.016). Model 3, an exploratory integration of the revised CAPL (Survey 1) and self-determination theory (Survey 2) measures, provided a good fit to the data (see Table 2).

Model 4 comprised an even shorter version of Model 3 (i.e., composite scores of intrinsic motivation, perceived competence satisfaction, adequacy, predilection). Model 4 was designed to integrate the revised CAPL (Survey 1) and self-determination theory (Survey 2) items while minimizing participant burden. To create this model, we dropped the new subscale we had labelled “behaviour”, as theoretically it might be tapping a behavioural component that is already assessed in the CAPL-2 via self-report physical activity and pedometer step counts. Further, we shortened each latent variable to have three items each. For the perceived competence satisfaction subscale, this was achieved by selecting the three items with the strongest factor loadings (i.e., “when it comes to playing active games, I think I am pretty good”, “I think I do well compared to other children”, and “when it comes to being active, I have good skills”). For the revised adequacy subscale, we dropped one item that was conceptually tapping similar content to the other items (i.e., “some kids think they are the best at sports BUT other kids think they aren’t good at sports”). Model 4 provided a superior fit to the data compared to the other models (see Table 2). Consequently, it was selected as the final model (see Additional file 6 for the final Motivation and Confidence domain questionnaire).

Three additional exploratory models containing various combinations of the Motivation and Confidence composite items were explored, but they are not presented here because they either did not provide a good fit, or were not closely aligned to Whitehead’s definition of motivation and confidence (see Additional file 7, Table 1 for results).

### Step 3: Revised motivation and confidence domain within the CAPL-2 model

Lastly, we ran a confirmatory factor analysis to examine the fit of the revised Motivation and Confidence domain in a four-correlated factor model representing all of the protocols within CAPL-2. In this model, a latent factor representing daily behaviour, comprising self-reported physical activity and daily step counts, could not be estimated because the two items were uncorrelated (*r* = − 0.03, *p* = 0.77). Therefore, daily step count was entered as an observed variable in the overall factor analytic model. Results of the four-correlated factor model indicated that the model was an excellent fit to the data (MLRχ^2^_(60)_ = 66.30, *p* = 0.27, CFI = 0.969, RMSEA = 0.023, 90% CI [0.00, 0.050]). The Knowledge and Understanding indictor asking “how to improve sport skill” did not significantly load onto knowledge and understanding (λ = 0.19, *p* = 0.13). All other factor loadings were significant (λ = 0.30–.92, *p*s < 0.05). Daily step count was not significantly correlated with any CAPL domain (*p*s > 0.14). Physical Competence was correlated with Knowledge and Understanding (*r* = 0.43, *p* < 0.001) and Motivation and Confidence (*r* = 0.29, *p* = 0.002). Motivation and Confidence was uncorrelated with knowledge and understanding (*r* = 0.14, *p* = 0.25).

**Fig. 1:**
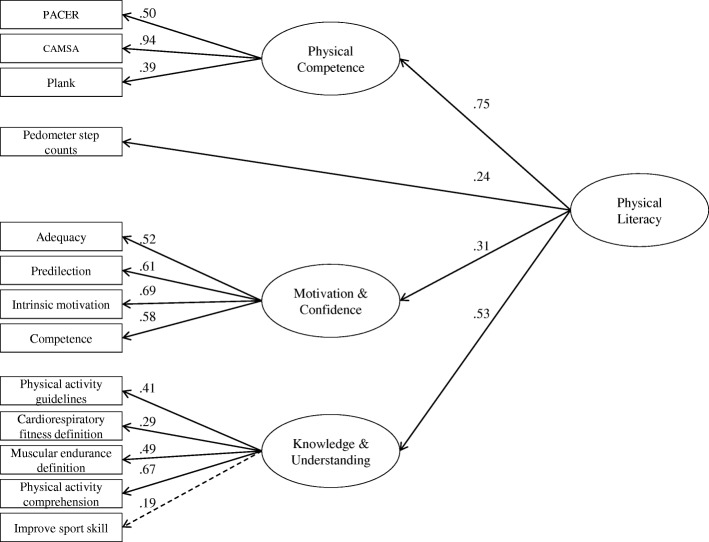
Higher-order confirmatory factor analysis of CAPL-2 with revised motivation and confidence domain. *Note.* Solid lines are statistically significant at *p* < 0.05; dashed line is statistically non-significant (*p* > 0.05). CAMSA: Canadian Agility and Movement Skill Assessment; PACER: Progressive Aerobic Cardiovascular Endurance Run

Next, this model was re-estimated specifying the four domains of CAPL-2 to load onto a single physical literacy latent factor. Results indicated a good model fit (MLRχ^2^_(63)_ = 81.45, *p* = 0.06, CFI = 0.908, RMSEA = 0.038, 90% CI [0.00, 0.060]; see Fig. 1). In a sensitivity analysis, this model was re-estimated omitting the 33 participants who completed questionnaires that used slightly different wording; the model fit (MLRχ^2^_(63)_ = 74.88, *p* = 0.15, CFI = 0.927, RMSEA = 0.033, 90% CI [0.00, 0.059]), and parameter estimates in the re-estimated model were very similar to when these participants were included (results available from Katie E. Gunnell upon request).

## Discussion

The purpose of this study was to further refine the Motivation and Confidence domain within the CAPL-2. We achieved our goal by reducing the domain from 36 single items to 12 single items which aggregate to four subscales. We retained two components of the original CAPL Motivation and Confidence assessment, albeit as much shorter versions. Additionally, we added two brief measures based on self-determination theory. As such, the revised Motivation and Confidence domain within CAPL-2 is theoretically anchored, contains clearer items, reduces burden on children, and has good score reliability and validity evidence based on factor structure (see Additional file 6).

Although scores from the benefits and barriers questionnaire demonstrated good factor structure after an error covariance was added, we did not include these items in our final revised domain of Motivation and Confidence. Our rationale stems from a conceptual and practical standpoint. First, as discussed in the introduction, benefits and barriers items might be more distal cognitive appraisals of motivation rather than proximal items aligned with Whitehead’s definition within a physical literacy context. Second, CAPL administrators identified that the barriers items were difficult for children to comprehend given their use of double negatives. Without further qualitative investigation, we were unable to determine if these items were indeed problematic in this sample of children. As such, future research is warranted to investigate response processes related to these items. Finally, we omitted the single item querying self-perceived skill compared to others. Although this item assesses confidence, it was on a Likert response format that was incompatible with the other Likert response questionnaires (i.e., had a 10-point scale rather than 5-point scale), thereby breaking a responding pattern children were familiar with in the other questionnaires. Furthermore, having only one item of confidence is limiting when researchers are seeking to perform factor analyses. Therefore, a decision was made to replace this one item with three items of perceived competence satisfaction [19] from an instrument that was developed based on theory and adapted for children using a similar response format to the intrinsic motivation measure.

Our confirmatory factor analysis of the alternative response scores from the original CAPL adequacy and predilection scores indicated that these scores alone did not provide a good fit to the data. Modification indices suggested cross-loadings as well as numerous correlated errors. These alternative response items were taken from the Children’s Self-Perceptions of Adequacy and Predilection for Physical Activity Scale [8], for which correlated errors have been reported in the Spanish version [32]. Given previous reports that the structured alternative response format could create method effects [12], we conducted an exploratory factor analysis to further investigate the factor structure of the alternative response items. We found evidence for a four-factor solution that we further narrowed down (based on the factor loadings and consideration of content representation) to three meaningful factors. To further reduce participant burden and also to reduce content overlap with other domains within CAPL, three items were selected for each of predilection and adequacy. These short measures of adequacy and predilection provided an excellent fit.

Consistent with the findings of Sebire and colleagues [19], we found that the factor structure of scores from the children’s adapted Behavioural Regulation in Exercise Questionnaire and five positively worded perceived competence satisfaction items were a good fit. Nevertheless, we also found that score reliability was low for introjected and external regulation. This finding is consistent with past self-determination theory-based research [20, 33], with some researchers speculating that younger children may not have sufficiently developed self-perception to be able to differentiate the more controlled reasons for behaviours. More research is needed to test the tenets of self-determination theory to determine if extrinsic and introjected regulations are salient and/or developmentally appropriate for young children, or if the current findings are obfuscated by measurement issues. Such research would lead to advancement in theory and may lead to future revisions and improvements in CAPL.

There were a few findings that were inconsistent with past research and theory. First, daily step counts were not associated with motivation and confidence, or any other domain of the CAPL. This finding is inconsistent with past research [5, 20], as well as with conceptualizations of physical literacy as interrelated domains [34] and tenets of self-determination theory [10]. It is possible that step counts were not significantly related to the domains of physical literacy as specified within the CAPL because children in this sample were highly active, as evident by high step count scores. More research is needed to determine if these null correlations are attributable to sample specific variation, sample size, or instrumentation (e.g., self-report, pedometer, or accelerometer assessments). Second, knowledge and understanding was unrelated to other CAPL domains except for one significant correlation with physical competence. This finding is inconsistent with the physical literacy consensus statement [34], yet consistent with other research that has given lower relative weight to this domain [9, 22]. Recently, Keegan and colleagues [11] hypothesized that physical literacy researchers often use quizzes to test specific aspects of explicit knowledge (e.g., knowledge of physical activity guidelines) rather than to also examine implicit beliefs that should be adopted for physical literacy. It may be worth further investigation into the Knowledge and Understanding domain of the CAPL to determine if the assessment is robust enough or if it requires modifications to capture explicit and implicit knowledge/beliefs. Alternatively, it is possible that knowledge and understanding is a more distal indicator of physical literacy in young children. Longmuir and Tremblay [35] recently suggested that more research is needed to determine if knowledge and understanding (and motivation and confidence) are salient for younger children (whose parents or social networks might dictate participation in physical activity) compared to older children (who have more autonomy).

### Limitations and future directions

Although we were able to refine the motivation and confidence assessments within CAPL, limitations are worth noting. First, the sample size was relatively small and we estimated numerous models, which could increase the odds of chance findings. Therefore, and in recognition that validation is an ongoing process, researchers should continue to replicate these finding with larger and more generalizable samples. Additionally, it is incumbent upon researchers who adopt these questionnaires to ensure that they demonstrate good score reliability and validity in their own samples before making inferences based on the data. Our sample might not generalize to other children since they were a select group of children participating in camps at YMCA. For example, it is possible that these children were more likely to be active than children who might have been recruited through other avenues; their parents may have prioritized physical activity more than other parents who did not enroll their children in the camps; or they could have come from lower socioeconomic status given that the YMCA offers physical activity programming for free.

Moreover, we were unable to model the Daily Behaviour domain comprised of both items of daily step counts and self-report physical activity because the two items were uncorrelated in this sample. Although weak correlations between pedometers and self-report physical activity have been noted in previous reviews [36], it was surprising to find no correlation between the two items in our sample. It is possible that the findings could be attributed to the unique sample in that they were children who were attending summer camps and whose activity was therefore similar throughout most of each day. Indeed, children in our sample had unusually high amounts of daily steps (M = 14,781, SD = 4244).

Although we were able to provide score validity and reliability evidence for our final selected model, other sources of validity should also be examined. The revised questionnaire to assess motivation and confidence in CAPL-2 comprises four subscales that include two response formats: namely, the structured alternative response format used in the adequacy and predilection sections, and the Likert-type response formats used in the intrinsic motivation and perceived competence satisfaction measures. Both formats have been criticized in previous literature for being difficult for children to understand [12, 17]. It is imperative that researchers interested in motivation and confidence in children further investigate these issues using techniques to understand how and why children are responding to these questionnaires the way they are. For example, an important next step for researchers is to examine validity evidence based on response processes [37] to further understand how and why children respond to the Likert and structured alternative response formats used to assess motivation and confidence.

Finally, our goal was to reduce the total number of items used to measure motivation and confidence. This, of course, comes at the cost of potentially reducing content representation and reliability. Researchers may wish to further investigate these issues to ensure that the items selected have good content validity evidence and reliability.

## Conclusions

Based on the findings from this study, we propose a revised questionnaire to assess motivation and confidence as part of the CAPL-2. The revised questionnaire is reduced to 12 single items that aggregate to four subscales, contains clearer instructions, and is theoretically aligned with a major theory of motivation. Researchers using the CAPL-2 should use the revised motivation and confidence questionnaire presented herein and presented in Additional file 4.

## Additional files

Additional file 1: Survey 1, original CAPL Motivation and Confidence questions. (DOCX 84 kb)

Additional file 2: Survey 2, new CAPL Motivation and Confidence questions. (DOCX 167 kb)

Additional file 3: Modified wording to new CAPL Motivation and Confidence questions. (DOCX 16 kb)

Additional file 4: Item descriptive statistics. (DOCX 34 kb)

Additional file 5: Model syntax. (DOCX 23 kb)

Additional file 6: Final Motivation and Confidence domain questionnaire. (DOCX 86 kb)

Additional file 7: Exploratory models. (DOCX 16 kb)

## Abbreviations

CAPL: Canadian Assessment of Physical Literacy
CAPL-1: Canadian Assessment of Physical Literacy, First Edition
CAPL-2: Canadian Assessment of Physical Literacy, Second Edition
CFI: comparative fit index
MLR: robust maximum likelihood
RMSEA: root mean square error of approximation
WLSMV: mean- and variance-adjusted weighted least square
