# Supplementary material for: Revising the motivation and confidence domain of the Canadian assessment of physical literacy
Source: BMC Public Health. 2018 Oct 2;18(Suppl 2):1045. doi: 10.1186/s12889-018-5900-0 (PMC6167763; doi:10.1186/s12889-018-5900-0)
Supplement: Supplementary file 1 — Survey 1, original CAPL Motivation and Confidence questions. (DOCX 84 kb) [file 12889_2018_5900_MOESM1_ESM.docx]

ID: _______________

**What’s Most Like Me**

For the rest of the questions you have to read two sentences and then circle the sentence you think is **MORE LIKE YOU**.

Try the following **SAMPLE QUESTION**:

| **Some kids have one nose on their face!** | **BUT** | **Other kids have three noses on their face!** |
| --- | --- | --- |

That shouldn’t be too hard for you to decide! Once you have circled the sentence that is more like you, then you have to decide if it is **REALLY TRUE** for you or **SORT OF TRUE** for you.

Here is another sample question for you to try. Remember, to answer the question you need to do two things:

1. **First circle the sentence that is more like you.**
2. **Then put a check in the correct box if it is really true or only sort of true for you**.

THERE ARE NO RIGHT OR WRONG ANSWERS, JUST WHAT IS **MOST LIKE YOU**.

**SAMPLE QUESTION #2**:

| **Some kids like to play with computers** | | **BUT** | **Other kids don’t like playing with computers** | |
| --- | --- | --- | --- | --- |
| **□ REALLY TRUE for me** | **□ SORT OF TRUE for me** |  | **□ REALLY TRUE for me** | **□ SORT OF TRUE for me** |

Now you are ready to start filling in this form**. Remember, in each box you need to circle what is most like you and then check a box for “really” or “sort of” true**. Take your time and do the whole form carefully. If you have any questions, just ask! If you think you are ready you can start now.

BE SURE TO FILL IN EACH PAGE!

**What’s Most Like Me?**

| **Some kids can’t wait to play active games after school** | | **BUT** | **Other kids would rather do something else after school** | |
| --- | --- | --- | --- | --- |
| **□ REALLY TRUE  for me** | **□ SORT OF TRUE  for me** |  | **□ REALLY   for me** | **□ SORT OF   for me** |

| **Some kids don’t like playing active games** | | **BUT** | **Other kids really like playing active games** | |
| --- | --- | --- | --- | --- |
| **□ REALLY   for me** | **□ SORT OF   for me** |  | **□ REALLY   for me** | **□ SORT OF   for me** |

| **Some kids don’t have much fun playing sports** | | **BUT** | **Other kids have a good time playing sports** | |
| --- | --- | --- | --- | --- |
| **□ REALLY TRUE   for me** | **□ SORT OF TRUE   for me** |  | **□ REALLY TRUE   for me** | **□ SORT OF TRUE   for me** |

| **Some kids are good at active games** | | **BUT** | **Other kids find active games hard to play** | |
| --- | --- | --- | --- | --- |
| **□ REALLY TRUE   for me** | **□ SORT OF TRUE   for me** |  | **□ REALLY TRUE   for me** | **□ SORT OF TRUE   for me** |

| **Some kids don’t like playing sports** | | **BUT** | **Other kids really enjoy playing sports** | |
| --- | --- | --- | --- | --- |
| **□ REALLY TRUE   for me** | **□ SORT OF TRUE   for me** |  | **□ REALLY TRUE   for me** | **□ SORT OF TRUE   for me** |

| **Some kids like to play active games outside** | | **BUT** | **Other kids would rather read or play video games** | |
| --- | --- | --- | --- | --- |
| **□ REALLY TRUE   for me for me** | **□ SORT OF TRUE   for me for me** |  | **□ REALLY TRUE   for me for me** | **□ SORT OF TRUE   for me for me** |

| **Some kids do well in most sports** | | **BUT** | **Other kids feel they aren’t good at sports** | |
| --- | --- | --- | --- | --- |
| **□ REALLY TRUE   for me** | **□ SORT OF TRUE   for me** |  | **□ REALLY TRUE   for me** | **□ SORT OF TRUE   for me** |

| **Some kids learn to play active games easily** | | **BUT** | **Other kids find it hard learning to play active games** | |
| --- | --- | --- | --- | --- |
| **□ REALLY TRUE   for me** | **□ SORT OF TRUE   for me** |  | **□ REALLY TRUE   for me** | **□ SORT OF TRUE   for me** |

| **Some kids think they are the best at sports** | | **BUT** | **Other kids think they aren’t good at sports** | |
| --- | --- | --- | --- | --- |
| **□ REALLY TRUE   for me** | **□ SORT OF TRUE   for me** |  | **□ REALLY TRUE   for me** | **□ SORT OF TRUE   for me** |

| **Some kids find games in physical education hard to play** | | **BUT** | **Other kids are good at games in physical education** | |
| --- | --- | --- | --- | --- |
| **□ REALLY TRUE   for me** | **□ SORT OF TRUE   for me** |  | **□ REALLY TRUE   for me** | **□ SORT OF TRUE   for me** |

| **Some kids like to watch games being played outside** | | **BUT** | **Other kids would rather play active games outside** | |
| --- | --- | --- | --- | --- |
| **□ REALLY TRUE   for me** | **□ SORT OF TRUE   for me** |  | **□ REALLY TRUE   for me** | **□ SORT OF TRUE   for me** |

| **Some kids are among the last to be chosen for active games** | | **BUT** | **Other kids are usually picked to play first** | |
| --- | --- | --- | --- | --- |
| **□ REALLY TRUE   for me** | **□ SORT OF TRUE   for me** |  | **□ REALLY TRUE   for me** | **□ SORT OF TRUE   for me** |

| **Some kids like to take it easy during recess** | | **BUT** | **Other kids would rather play active games at recess** | |
| --- | --- | --- | --- | --- |
| **□ REALLY TRUE   for me** | **□ SORT OF TRUE   for me** |  | **□ REALLY TRUE   for me** | **□ SORT OF TRUE   for me** |

| **Some kids aren’t good enough for sports teams** | | **BUT** | **Other kids do well on sports teams** | |
| --- | --- | --- | --- | --- |
| **□ REALLY TRUE   for me** | **□ SORT OF TRUE   for me** |  | **□ REALLY TRUE   for me** | **□ SORT OF TRUE   for me** |

| **Some kids like to read or play quiet games** | | **BUT** | **Other kids like to play active games** | |
| --- | --- | --- | --- | --- |
| **□ REALLY TRUE   for me** | **□ SORT OF TRUE   for me** |  | **□ REALLY TRUE   for me** | **□ SORT OF TRUE   for me** |

| **Some kids like to play active games outside on weekends** | | **BUT** | **Other kids like to relax and watch TV on weekends** | |
| --- | --- | --- | --- | --- |
| **□ REALLY TRUE   for me** | **□ SORT OF TRUE   for me** |  | **□ REALLY TRUE   for me** | **□ SORT OF TRUE   for me** |

**Thank you for telling us how you feel!**

We have just a few more questions about physical activity.

Please turn to the next page.

2. Kids say there are many different reasons that they like to be active or play sports. Being active is anything that you do when you are moving, exercising or not sitting still. Below are some reasons that other kids have told us why they like to be active. For each reason,
**tell us what you think by** **choosing the number that shows how you feel**.

5 means you think it is a really, really good reason to be active. You **totally agree** with other kids who say this is a good reason to be active.

4 means you think it is a pretty good reason to be active. You **agree** with other kids who say this is a good reason to be active.

3 means you are not sure or you don’t think the reason is good or bad. Choose this answer if you are **in between**.

2 means you think it is not a good reason to be active. You **disagree** with other kids who say this is a good reason to be active.

1 means you think it is a really, really bad reason to be active. You **totally disagree** with other kids who say this is a good reason to be active.

| A reason that I might be active is because when I am active. . . | Disagree  a lot | Disagree  a little | In Between | Agree  a little | Agree a lot |
| --- | --- | --- | --- | --- | --- |
| …I look better | 1 | 2 | 3 | 4 | 5 |
| … I have more energy | 1 | 2 | 3 | 4 | 5 |
| …I feel happier | 1 | 2 | 3 | 4 | 5 |
| …I have fun | 1 | 2 | 3 | 4 | 5 |
| … I make more friends | 1 | 2 | 3 | 4 | 5 |
| …I get stronger | 1 | 2 | 3 | 4 | 5 |
| … I like myself more | 1 | 2 | 3 | 4 | 5 |
| …I get in better shape | 1 | 2 | 3 | 4 | 5 |
| …I feel healthier | 1 | 2 | 3 | 4 | 5 |

3. Kids say there are also reasons that make it hard for them to be active. For each reason, **tell us what you think**. If you think it is a good reason then you would “Agree a little” or “Agree a lot”. If you do not think it’s a good reason, then you would “Disagree a little” or “Disagree a lot”. If you are not sure or you don’t think the reason is good or bad then you are “in between”.

5 means you think it is a really, really good reason for not being active. You **totally agree** with other kids who say this is a good reason for not being active.

4 means you think it is a pretty good reason for not being active. You **agree** with other kids who say this is a good reason for not being active.

3 means you are not sure or you don’t think the reason is good or bad. Choose this answer if you are **in between**.

2 means you think it is not a good reason for not being active. You **disagree** with other kids who say this is a good reason for not being active.

1 means you think it is a really, really bad reason for not being active. You **totally disagree** with other kids who say this is a good reason for not being active.

| I might not be active if. . | Disagree  a lot | Disagree  a little | In Between | Agree  a little | Agree a lot |
| --- | --- | --- | --- | --- | --- |
| …I didn’t have enough  time to be active | 1 | 2 | 3 | 4 | 5 |
| …I have too many chores to do | 1 | 2 | 3 | 4 | 5 |
| …I didn’t have a good place to be active | 1 | 2 | 3 | 4 | 5 |
| …If the weather was too  bad | 1 | 2 | 3 | 4 | 5 |
| …I didn’t have the right  clothes/shoes | 1 | 2 | 3 | 4 | 5 |
| …I didn’t know how to  do the activity | 1 | 2 | 3 | 4 | 5 |
| …I didn’t have the right  equipment | 1 | 2 | 3 | 4 | 5 |
| …I had too much  homework | 1 | 2 | 3 | 4 | 5 |
| …I didn’t have anyone to  be active with | 1 | 2 | 3 | 4 | 5 |
| …I didn’t like to be active | 1 | 2 | 3 | 4 | 5 |

☺ Thank you for answering our questions! ☺
