# Supplementary material for: Revising the motivation and confidence domain of the Canadian assessment of physical literacy
Source: BMC Public Health. 2018 Oct 2;18(Suppl 2):1045. doi: 10.1186/s12889-018-5900-0 (PMC6167763; doi:10.1186/s12889-018-5900-0)
Supplement: Supplementary file 2 — Survey 2, new CAPL Motivation and Confidence questions. (DOCX 167 kb) [file 12889_2018_5900_MOESM2_ESM.docx]

ID: ___________________

1. What school grade are you in: (please circle one)
   If you are not in school today, please circle the grade you will be in on the next day that you will go to school.

1 2 3 4 5 6 7 8

1. Are you a: boy girl (please circle one)
2. What month is your birthday: (please circle one)

Jan Feb Mar Apr May Jun Jul Aug Sept Oct Nov Dec

1. How old are you: (please circle one)

5 6 7 8 9 10 11 12 13 14 15

When you read questions about physical activity, we mean when you are moving around, playing or exercising. Physical activity is any activity that makes your heart beat faster or makes you get out of breath some of the time.

Why are we asking you these questions? We want to know what kids like you think about physical activity, sports and exercise.

Please remember:


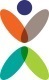
 There are no right or wrong answers! We only want to know what you think.


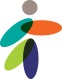
 If you do not know an answer, please write your best guess.


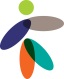
 There is no time limit, so please take all the time you need.

**Why are you active?**

Boys and girls can be **active** by doing all sorts of things:

- Exercise (walking, keeping fit, or gym class)
- Playing outside or doing active things (like playing in the park)
- Sports (like soccer, tennis, hockey, dance or swimming)

Below are some reasons why you might be active.

Please read each sentence and tell us how true it is for you.

| **I am active because...** | | | | | | |
| --- | --- | --- | --- | --- | --- | --- |
|  |  | Not true for me | Not really true for me | Sometimes true for me | Often true for me | Very true for me |
| 1. | being active is fun | € | € | € | € | € |
| 2. | it is important to me to do active things | € | € | € | € | € |
| 3. | when I’m not active I feel bad | € | € | € | € | € |
| 4. | other people say I should be active | € | € | € | € | € |
| 5. | I enjoy being active | € | € | € | € | € |
| 6. | I value the benefits of being active | € | € | € | € | € |
| 7. | when I don’t do activity I feel bad about myself | € | € | € | € | € |
| 8. | if I’m not active, other people will not be pleased with me | € | € | € | € | € |
| 9. | I like being active | € | € | € | € | € |
| 10. | in life it is important to be active | € | € | € | € | € |
| 11. | I want to show other people how good I am at being active | € | € | € | € | € |
| 12. | other people pressure me to be active | € | € | € | € | € |


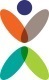


**How do you feel about being active?**

The next section has some sentences describing how girls and boys feel about BEING ACTIVE and DOING ACTIVE THINGS (like active games, playing outside and doing sports).

Please read each sentence and tell us how much each sentence is like you.

|  |  | Not like me at all | Not really like me | Sometimes like me | Quite a lot like me | Really like me |
| --- | --- | --- | --- | --- | --- | --- |
| 1. | When it comes to playing active games, I think I am pretty good. | € | € | € | € | € |
| 2. | I think I do well at activities compared to other children | € | € | € | € | € |
| 3. | After working at a new activity for a while, I feel that I can do it pretty well. | € | € | € | € | € |
| 4. | I am happy with how good I am at doing active games. | € | € | € | € | € |
| 5. | When it comes to being active, I have good skills. | € | € | € | € | € |
| 6. | I can’t do physical activities very well. | € | € | € | € | € |

**Thank you for telling us how you feel!**

We have just a few more questions about physical activity.

Please turn to the next page.


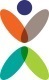
1. How many minutes each day should you and other children do physical activities that make your heart beat faster and make you breathe faster, like walking fast or running? Count the time you should be active at school and also when you are at home or in your neighbourhood.

a) 20 minutes

b) 30 minutes

c) 60 minutes or 1 hour

d) 120 minutes or 2 hours

2. There are many different kinds of fitness. One type is called endurance fitness or aerobic fitness or cardiorespiratory fitness. Cardiorespiratory fitness means…
(circle the right answer)

a) How well the muscles can push, pull or stretch.

b) How well the heart can pump blood and the lungs can provide oxygen.

c) Having a healthy weight for our height.

d) Our ability to do sports that we like.

3. Muscular strength or muscular endurance means…
(circle the right answer)

a) How well the muscles can push, pull or stretch.

b) How well the heart can pump blood and the lungs can provide oxygen.

c) Having a healthy weight for our height.

d) Our ability to do sports that we like.

4. If you wanted to GET BETTER AT A SPORT SKILL like kicking and catching a ball, what would be the best thing to do?
(circle one answer)

a) Read a book about kicking and catching a ball

b) Wait until you get older

c) Try exercising or being active a lot more

d) Watch a video, take a lesson or have a coach teach you how to kick and catch

5. Compared to other kids your age, how good are you at sports or skills? (circle one number)

Others are better Same I’m a lot better

1 2 3 4 5 6 7 8 9 10

6. This story about Sally is missing some words. Choose from the words in the box to fill in the missing words in the story. Each word can only be used to fill one blank space in the story. There are more words than blank spaces, so not all words will be used.


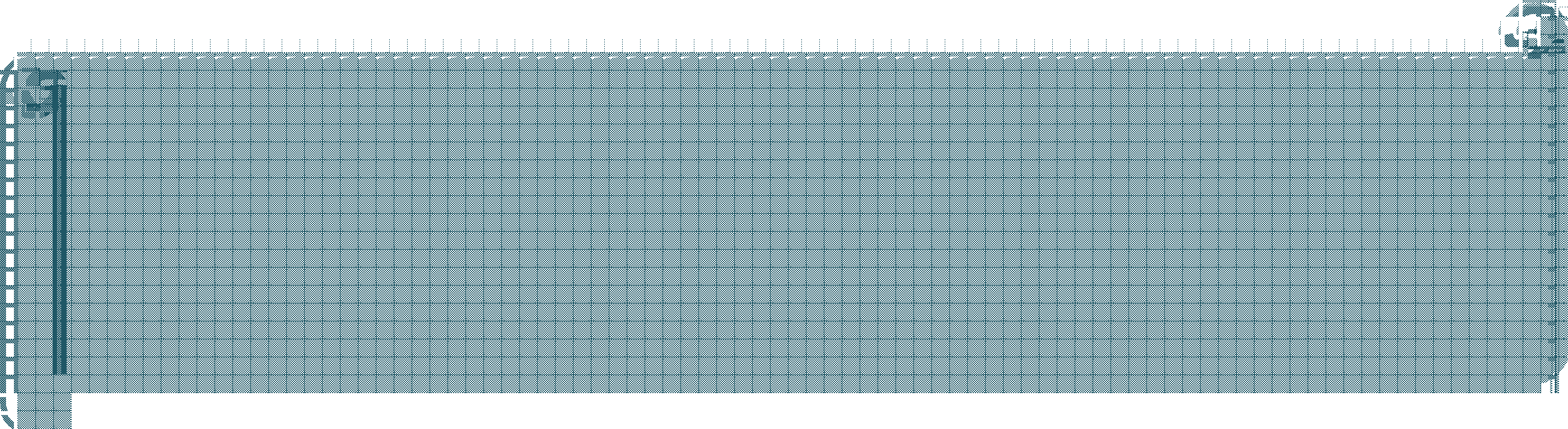


Fun Stretches Endurance
Breathing Flexibility Good

Pulse Strength Bad Balance

Sally tries to be active every day. Running every day is good for her heart and lungs.

Sally thinks that physical activity is and is also for her. At her sport team’s practice she does more running to improve her _. The team also does exercises like push-ups and sit-ups that increase her . When cooling down, she ______________ to improve her flexibility and lower her heart rate. After exercising, she checks her heart rate which is also called a _.

7. During the past week (7 days), on how many days were you physically active for a total of at least 60 minutes per day? (count all of the time you spent doing activities that increased your heart rate or made you breathe hard)

I was active for 0 1 2 3 4 5 6 7 days

☺ Thank you for answering our questions! ☺
