# Supplementary material for: Revising the motivation and confidence domain of the Canadian assessment of physical literacy
Source: BMC Public Health. 2018 Oct 2;18(Suppl 2):1045. doi: 10.1186/s12889-018-5900-0 (PMC6167763; doi:10.1186/s12889-018-5900-0)
Supplement: Supplementary file 3 — Modified wording to new CAPL Motivation and Confidence questions. (DOCX 16 kb) [file 12889_2018_5900_MOESM3_ESM.docx]

**Additional File 3**

**Table S1.**

Child adapted Behavioral Regulation in Exercise Questionnaire Modified wording

| Item number | Subscale | Item wording |
| --- | --- | --- |
| 1. | Intrinsic | being active is fun |
| 2. | Identified | it is important to me to do active things |
| 3. | Introjected | when I’m not active I feel bad |
| 4. | Extrinsic | other people say I should be **active** |
| 5. | Intrinsic | I enjoy being active |
| 6. | Identified | I value the benefits of being active |
| 7. | Introjected | when I don’t do activity I feel bad about myself |
| 8. | Extrinsic | if I’m not **active**, other people will not be pleased with me |
| 9. | Intrinsic | I like being active |
| 10. | Identified | in life it is important to be active |
| 11. | Introjected | I want to show other people how good I am **at being active** |
| 12. | Extrinsic | other people pressure me to be active |

Note. Bolded words were added to the original items from Sebire et al., (2013) to enhance clarity.

**Table S2.**

Child adapted Perceived Competence Satisfaction Modified wording

| Item Number | Item |
| --- | --- |
| 1. | When it comes to playing active games, I think I am pretty good. |
| 2. | I think I do well **at activities** compared to other children |
| 3. | After working at a new activity for a while, I feel that I can do it pretty well. |
| 4. | I am happy with how good I am at doing active games. |
| 5. | When it comes to being active, I have good skills. |
| 6. | I can’t do physical activities very well. |

Note. Bolded words were added to the original items from Sebire et al., (2013) to enhance clarity.
