# Supplementary material for: Revising the motivation and confidence domain of the Canadian assessment of physical literacy
Source: BMC Public Health. 2018 Oct 2;18(Suppl 2):1045. doi: 10.1186/s12889-018-5900-0 (PMC6167763; doi:10.1186/s12889-018-5900-0)
Supplement: Supplementary file 4 — Item descriptive statistics. (DOCX 34 kb) [file 12889_2018_5900_MOESM4_ESM.docx]

**Table S1**

Predilection and Adequacy Items

|  | | | | | | | |
| --- | --- | --- | --- | --- | --- | --- | --- |
|  | Mean | SD | Skewness | | Kurtosis | |  |
|  | Statistic | Statistic | Statistic | Std. Error | Statistic | Std. Error |  |
| Predilection_1 | 3.0049 | 1.10922 | -.728 | .170 | -.874 | .338 |  |
| Predilection _2 | 3.1512 | 1.12091 | -.914 | .170 | -.707 | .338 |  |
| Predilection _3 | 3.2390 | 1.01771 | -1.030 | .170 | -.279 | .338 |  |
| Adequacy_4 | 3.2537 | .89336 | -.981 | .170 | .027 | .338 |  |
| Predilection _5 | 3.2000 | 1.08646 | -1.031 | .170 | -.405 | .338 |  |
| Predilection _7 | 3.0780 | 1.11749 | -.815 | .170 | -.810 | .338 |  |
| Adequacy _8 | 2.7512 | 1.08083 | -.314 | .170 | -1.187 | .338 |  |
| Adequacy _9 | 3.2488 | .96078 | -1.120 | .170 | .190 | .338 |  |
| Adequacy _10 | 3.2683 | .90814 | -1.073 | .170 | .218 | .338 |  |
| Adequacy _11 | 2.8390 | .95919 | -.581 | .170 | -.534 | .338 |  |
| Adequacy _12 | 3.2976 | .93633 | -1.206 | .170 | .440 | .338 |  |
| Predilection _13 | 3.2927 | 1.05356 | -1.219 | .170 | .033 | .338 |  |
| Predilection _14 | 3.1024 | 1.13511 | -.833 | .170 | -.851 | .338 |  |
| Adequacy _15 | 3.0585 | 1.05556 | -.699 | .170 | -.856 | .338 |  |
| Predilection _16 | 3.1171 | 1.12283 | -.884 | .170 | -.729 | .338 |  |
| Predilection _17 | 2.8927 | 1.15393 | -.562 | .170 | -1.164 | .338 |  |
| Skill compared to others | 7.0585 | 2.08319 | -.456 | .170 | -.305 | .338 |  |

Note: Adequacy and predilection item values range from 1-4. Skill compared to others item ranges from 1-10. Final model used items Predilection_2, Predilectoin_3, prediliection_5 and adequacy_4, adequacy_9, adequacy_10.

**Table S2**

Benefits and Barriers Items

|  | | | | | | |
| --- | --- | --- | --- | --- | --- | --- |
|  | Mean | SD | Skewness | | Kurtosis | |
|  | Statistic | Statistic | Statistic | Std. Error | Statistic | Std. Error |
| benefit_look_better | 2.8439 | 1.48 | .053 | .170 | -1.367 | .338 |
| benefit_more_energy | 4.2780 | 1.06 | -1.449 | .170 | 1.409 | .338 |
| benefit_feel_happier | 4.2098 | 1.10 | -1.322 | .170 | .978 | .338 |
| benefit_have_fun | 4.6195 | .89 | -2.627 | .170 | 6.566 | .338 |
| benefit_make_more_friends | 4.0537 | 1.21 | -1.094 | .170 | .140 | .338 |
| benefit_get_stronger | 4.4976 | .93 | -2.306 | .170 | 5.377 | .338 |
| benefit_like_myself_more | 3.3707 | 1.55 | -.382 | .170 | -1.346 | .338 |
| benefit_like_get_in_better_shape | 4.2829 | 1.10 | -1.593 | .170 | 1.845 | .338 |
| benefit_like_feel_healthier | 4.5317 | .94 | -2.313 | .170 | 5.123 | .338 |
| barrier_didnt_have_time | 2.1756 | 1.37 | .856 | .170 | -.581 | .338 |
| barrier_too_many_chores | 2.2146 | 1.27 | .747 | .170 | -.454 | .338 |
| barrier_didnt_have_good_place_tobe_active | 2.0732 | 1.39 | 1.006 | .170 | -.344 | .338 |
| barrier_weather_too_bad | 2.9902 | 1.57 | .001 | .170 | -1.527 | .338 |
| barrier_didnt_have_right_clothes | 2.0829 | 1.34 | 1.023 | .170 | -.166 | .338 |
| barrier_didnt_know_how_to_do_activity | 2.0878 | 1.29 | .864 | .170 | -.489 | .338 |
| barrier_didnt_have_right_equipment | 2.1805 | 1.32 | .821 | .170 | -.515 | .338 |
| barrier_too_much_homework | 2.5659 | 1.48 | .422 | .170 | -1.202 | .338 |
| barrier_didnt_have_anyone_tobe_activewith | 2.0683 | 1.35 | 1.001 | .170 | -.264 | .338 |
| barrier_didnt_like_to_be_active | 1.5561 | 1.19 | 2.015 | .170 | 2.677 | .338 |

Note: values range from 1-5.

**Table S3**

Motivational Regulation Items

|  | Mean | SD | Skewness | | Kurtosis | |
| --- | --- | --- | --- | --- | --- | --- |
|  | Statistic | Statistic | Statistic | Std. Error | Statistic | Std. Error |
| Intrinsic_1 | 4.3707 | .85699 | -1.173 | .170 | .621 | .338 |
| Identified_2 | 4.3512 | .89309 | -1.211 | .170 | .648 | .338 |
| Introjected_3 | 2.9659 | 1.39460 | .018 | .170 | -1.204 | .338 |
| Extrinsic_4 | 2.6732 | 1.54524 | .335 | .170 | -1.393 | .338 |
| Intrinsic _5 | 4.5073 | .82616 | -1.604 | .170 | 1.876 | .338 |
| Identified _6 | 4.0927 | 1.07840 | -.920 | .170 | -.039 | .338 |
| Introjected _7 | 2.6780 | 1.43614 | .269 | .170 | -1.262 | .338 |
| Extrinsic _8 | 1.9756 | 1.26584 | 1.101 | .170 | .042 | .338 |
| Intrinsic _9 | 4.5024 | .91085 | -1.914 | .170 | 3.282 | .338 |
| Identified _10 | 4.4878 | .88898 | -1.802 | .170 | 2.866 | .338 |
| Introjected _11 | 3.3268 | 1.43674 | -.339 | .170 | -1.177 | .338 |
| Extrinsic _12 | 2.0585 | 1.33449 | 1.054 | .170 | -.125 | .338 |

Note: values range from 1-5. Final model used items 1, 5, 9.

**Table S4**

Perceived Competence Items

|  | | | | | | |
| --- | --- | --- | --- | --- | --- | --- |
|  | Mean | SD | Skewness | | Kurtosis | |
|  | Statistic | Statistic | Statistic | Std. Error | Statistic | Std. Error |
| Perceived Competence_1 | 4.0537 | 1.04415 | -.943 | .170 | .326 | .338 |
| Perceived Competence_2 | 3.7317 | 1.15522 | -.616 | .170 | -.362 | .338 |
| Perceived Competence_3 | 4.2439 | .99461 | -1.352 | .170 | 1.512 | .338 |
| Perceived Competence_4 | 4.5073 | .82616 | -1.604 | .170 | 1.876 | .338 |
| Perceived Competence_5 | 4.0829 | .95894 | -.706 | .170 | -.281 | .338 |
| Perceived Competence_6 | 1.7951 | 1.22353 | 1.420 | .170 | .810 | .338 |
| Perceived Competence_6_reverse coded | 4.2049 | 1.22353 | -1.420 | .170 | .810 | .338 |

Note: values range from 1-5. Final model used items 1, 2, 5.

**Table S5**

Descriptive Statistics for Physical Competence and Pedometer Scores

|  | N | Minimum | Maximum | Mean | SD | Skewness | | Kurtosis | |
| --- | --- | --- | --- | --- | --- | --- | --- | --- | --- |
|  | Statistic | Statistic | Statistic | Statistic | Statistic | Statistic | Std. Error | Statistic | Std. Error |
| PACER | 199 | 3.00 | 52.00 | 18.4171 | 10.75587 | 1.050 | .172 | .463 | .343 |
| Plank | 203 | 6.00 | 276.00 | 67.7187 | 46.02773 | 1.398 | .171 | 2.612 | .340 |
| CAMSA | 203 | 11.00 | 27.00 | 20.5271 | 3.55062 | -.300 | .171 | -.501 | .340 |
| Pedometer Step Counts | 107 | 4801.00 | 26412.00 | 14780.9720 | 4243.91523 | .133 | .234 | -.311 | .463 |

Note: CAMSA = Canadian Agility and Movement Skill Assessment, PACER = Progressive Aerobic Cardiovascular Endurance Run

**Table S6**

Descriptive Statistics for Knowledge and Understanding Variables

|  | Mean | Median | Mode | SD | Skewness | | Kurtosis | |
| --- | --- | --- | --- | --- | --- | --- | --- | --- |
|  | Statistic | Statistic | Statistic | Statistic | Statistic | Std. Error | Statistic | Std. Error |
| Cardiorespiratory Fitness Definition | .551 | 1 | 1 | .50 | -.207 | .170 | -1.976 | .338 |
| Muscular Endurance Definition | .678 | 1 | 1 | .47 | -.768 | .170 | -1.425 | .338 |
| Physical Activity Comprehension | 3.834 | 4 | 3 | 1.58 | -.176 | .170 | -.878 | .338 |
| Improve Sport Skill | .415 | 0 | 0 | .49 | .349 | .170 | -1.897 | .338 |
| Physical Activity Guidelines | .229 | 0 | 0 | .42 | 1.298 | .170 | -.319 | .338 |

Note: all values range from 0-1 except physical activity comprehension, which ranges from 0-6. A value of 1 represents “correct” and 0 represents “incorrect”.
