# Supplementary material for: Revising the motivation and confidence domain of the Canadian assessment of physical literacy
Source: BMC Public Health. 2018 Oct 2;18(Suppl 2):1045. doi: 10.1186/s12889-018-5900-0 (PMC6167763; doi:10.1186/s12889-018-5900-0)
Supplement: Supplementary file 5 — Model syntax. (DOCX 23 kb) [file 12889_2018_5900_MOESM5_ESM.docx]

# Reliability Formulas

## Coefficient H

Coefficient H formula:

H= $\frac{\sum_{i=1}^{k} \left( \frac{\mathcal{l}_{i}^{2}}{1-\mathcal{l}_{i}^{2}} \right)}{1+\sum_{i=1}^{k} \left( \frac{\mathcal{l}_{i}^{2}}{1-\mathcal{l}_{i}^{2}} \right)}$

Where 𝓁i is a standardized factor loading (Hancock & Mueller, 2001).

## Omega Total

$$\frac{(\sum\lambda)2}{\left( \Sigma\lambda\right)2+\Sigma\theta]}$$

Where λ is a standardized factor loading, and θ is a residual variance (Diamantopoulos, A., & Siguaw, J. (2000). Introducing LISREL. London: SAGE Publications Ltd.)

# Mplus Syntax for analyses in Step 1

## Adequacy and Predilection Items (CFA; Original)

VARIABLE:

NAMES ARE age age_dec sex pacer plank

camsa step3 step4 self_pa csappa1-csappa5

csappa7-csappa17 ben1-ben9 bar1-bar10

adeq_sum adeq_score pred_sum pred_score

skill_cmprag card_def musc_def pa_is pais_also

Krun Kpushup Kcool Kheart blank_score imprskil

PAQ breq1-breq12 comp1-comp6 comp6_r;

USEVARIABLES ARE

csappa1-csappa5

csappa7-csappa17;

MISSING ARE ALL (999.99);

ANALYSIS:

ESTIMATOR = MLR

MODEL:

PRED BY CSAPPA1-CSAPPA3 CSAPPA5 CSAPPA7

CSAPPA13 CSAPPA14 CSAPPA16 CSAPPA17;

ADEQ BY CSAPPA4 CSAPPA8-CSAPPA12 CSAPPA15;

output: STDYX tech4 modindices;

## Adequacy and Predilection Items (EFA)

VARIABLE:

NAMES ARE age age_dec sex pacer plank

camsa step3 step4 self_pa csappa1-csappa5

csappa7-csappa17 ben1-ben9 bar1-bar10

adeq_sum adeq_score pred_sum pred_score

skill_cmprag card_def musc_def pa_is pais_also

Krun Kpushup Kcool Kheart blank_score imprskil

PAQ breq1-breq12 comp1-comp6 comp6_r;

USEVARIABLES ARE

csappa1-csappa5

csappa7-csappa17;

MISSING ARE ALL (999.99);

ANALYSIS:

type = efa 1 5;

plot: type = plot2;

output: STDYX tech4 modindices;

## Adequacy and Predilection Items (CFA; New Model based on EFA)

VARIABLE:

NAMES ARE age age_dec pacer plank

camsa step3 step4 self_pa csappa1-csappa5

csappa7-csappa17 ben1-ben9 bar1-bar10

adeq_sum adeq_score pred_sum pred_score

skill_cmprag card_def musc_def pa_is pais_also

Krun Kpushup Kcool Kheart blank_score imprskil

PAQ breq1-breq12 comp1-comp6 comp6_r IM ID IJ

EX compt Ben Bar ratio;

USEVARIABLES ARE

csappa1-csappa5 csappa7 csappa17

csappa9 csappa10;

MISSING ARE ALL (999.99);

ANALYSIS:

ESTIMATOR = MLR;

MODEL:

doing by csappa1* csappa7 csappa17;

pred by csappa2* csappa3 csappa5;

Adeq by csappa4* csappa9 csappa10 csappa11;

Pred@1;

doing@1;

adeq@1;

output: STDYX sampstat tech4 modindices;

## Benefits and Barriers Scale (CFA Original)

VARIABLE:

NAMES ARE age age_dec sex pacer plank

camsa step3 step4 self_pa csappa1-csappa5

csappa7-csappa17 ben1-ben9 bar1-bar10

adeq_sum adeq_score pred_sum pred_score

skill_cmprag card_def musc_def pa_is pais_also

Krun Kpushup Kcool Kheart blank_score imprskil

PAQ breq1-breq12 comp1-comp6 comp6_r;

USEVARIABLES ARE

ben1-ben9

bar1-bar10;

MISSING ARE ALL (999.99);

ANALYSIS:

ESTIMATOR = MLR

MODEL:

ben by ben1* ben2-ben9;

ben@1;

bar by bar1* bar2-bar10;

bar@1;

output: STDYX tech4 modindices;

## Benefits and Barriers (CFA; Revised)

VARIABLE:

NAMES ARE age age_dec sex pacer plank

camsa step3 step4 self_pa csappa1-csappa5

csappa7-csappa17 ben1-ben9 bar1-bar10

adeq_sum adeq_score pred_sum pred_score

skill_cmprag card_def musc_def pa_is pais_also

Krun Kpushup Kcool Kheart blank_score imprskil

PAQ breq1-breq12 comp1-comp6 comp6_r;

USEVARIABLES ARE

ben1-ben9

bar1-bar10;

MISSING ARE ALL (999.99);

ANALYSIS:

ESTIMATOR = MLR

MODEL:

ben by ben1* ben2-ben9;

ben@1;

ben7 with ben1;

bar by bar1* bar2-bar10;

bar@1;

output: STDYX tech4 modindices;

## Perceived Competence (CFA; original)

VARIABLE:

NAMES ARE age age_dec sex pacer plank

camsa step3 step4 self_pa csappa1-csappa5

csappa7-csappa17 ben1-ben9 bar1-bar10

adeq_sum adeq_score pred_sum pred_score

skill_cmprag card_def musc_def pa_is pais_also

Krun Kpushup Kcool Kheart blank_score imprskil

PAQ breq1-breq12 comp1-comp6 comp6_r;

USEVARIABLES ARE

comp1-comp5 comp6_r;

MISSING ARE ALL (999.99);

ANALYSIS:

ESTIMATOR = MLR

MODEL:

comp by comp1* comp2-comp5 comp6_r;

comp@1;

output: STDYX tech4 modindices;

## Perceived Competence (CFA; Shortened)

VARIABLE:

NAMES ARE age age_dec sex pacer plank

camsa step3 step4 self_pa csappa1-csappa5

csappa7-csappa17 ben1-ben9 bar1-bar10

adeq_sum adeq_score pred_sum pred_score

skill_cmprag card_def musc_def pa_is pais_also

Krun Kpushup Kcool Kheart blank_score imprskil

PAQ breq1-breq12 comp1-comp6 comp6_r;

USEVARIABLES ARE

comp1-comp5;

MISSING ARE ALL (999.99);

ANALYSIS:

ESTIMATOR = MLR

MODEL:

comp by comp1* comp2-comp5;

comp@1;

output: STDYX tech4 modindices;

## Motivational Regulations (CFA; Original)

VARIABLE:

NAMES ARE age age_dec sex pacer plank

camsa step3 step4 self_pa csappa1-csappa5

csappa7-csappa17 ben1-ben9 bar1-bar10

adeq_sum adeq_score pred_sum pred_score

skill_cmprag card_def musc_def pa_is pais_also

Krun Kpushup Kcool Kheart blank_score imprskil

PAQ breq1-breq12 comp1-comp6 comp6_r;

USEVARIABLES ARE

breq1-breq12;

MISSING ARE ALL (999.99);

ANALYSIS:

ESTIMATOR = MLR

MODEL:

IM by breq1* breq5 breq9;

ID by breq2* breq6 breq10;

IJ by breq3* breq7 breq11;

EX by breq4* breq8 breq12;

im@1;

id@1;

IJ@1;

EX@1;

output: STDYX tech4 modindices;

# Mplus Syntax for analyses in Step 2

## Model 1 (Original CAPL model)

VARIABLE:

NAMES ARE age age_dec pacer plank

camsa step3 step4 self_pa csappa1-csappa5

csappa7-csappa17 ben1-ben9 bar1-bar10

adeq_sum adeq_score pred_sum pred_score

skill_cmprag card_def musc_def pa_is pais_also

Krun Kpushup Kcool Kheart blank_score imprskil

PAQ breq1-breq12 comp1-comp6 comp6_r IM ID IJ

EX comp Ben Bar ratio;

USEVARIABLES ARE

pred_sum adeq_sum

ratio skill_cmprag;

MISSING ARE ALL (999.99);

ANALYSIS:

ESTIMATOR = MLR

MODEL:

Motiv by pred_sum*

adeq_sum ratio skill_cmprag;

motiv@1;

output: STDYX sampstat tech4 modindices;

## Model 2 (Self-determination theory measures)

VARIABLE:

NAMES ARE age age_dec pacer plank

camsa step3 step4 self_pa csappa1-csappa5

csappa7-csappa17 ben1-ben9 bar1-bar10

adeq_sum adeq_score pred_sum pred_score

skill_cmprag card_def musc_def pa_is pais_also

Krun Kpushup Kcool Kheart blank_score imprskil

PAQ breq1-breq12 comp1-comp6 comp6_r IM ID IJ

EX comp Ben Bar ratio;

USEVARIABLES ARE

IM ID IJ EX COMP;

MISSING ARE ALL (999.99);

ANALYSIS:

ESTIMATOR = MLR

MODEL:

Motiv by IM* ID IJ EX COMP;

motiv@1;

output: STDYX sampstat tech4 modindices;

## Model 3 (Intrinsic regulation, skill compared to peers, shortened adequacy, shortened predilection and ‘behaviour’)

VARIABLE:

NAMES ARE age age_dec pacer plank

camsa step3 step4 self_pa csappa1-csappa5

csappa7-csappa17 ben1-ben9 bar1-bar10

adeq_sum adeq_score pred_sum pred_score

skill_cmprag card_def musc_def pa_is pais_also

Krun Kpushup Kcool Kheart blank_score imprskil

PAQ breq1-breq12 comp1-comp6 comp6_r IM ID IJ

EX comp Ben Bar ratio;

USEVARIABLES ARE

IM skill_cmprag behav pred adeq;

MISSING ARE ALL (999.99);

DEFINE:

behav = (csappA1+csappa7+Csappa17)/3;

pred = (csappa2+csappa3 + csappa5)/3;

adeq= (csappa4+csappa9+csappa10+csappa11)/4;

ANALYSIS:

ESTIMATOR = MLR

MODEL:

Motiv by IM* skill_cmprag behav pred adeq;

motiv@1;

output: STDYX sampstat tech4 modindices;

## Model 4 (Shortened version of Model 3)

VARIABLE:

NAMES ARE age age_dec pacer plank

camsa step3 step4 self_pa csappa1-csappa5

csappa7-csappa17 ben1-ben9 bar1-bar10

adeq_sum adeq_score pred_sum pred_score

skill_cmprag card_def musc_def pa_is pais_also

Krun Kpushup Kcool Kheart blank_score imprskil

PAQ breq1-breq12 comp1-comp6 comp6_r IM ID IJ

EX comp Ben Bar ratio;

USEVARIABLES ARE

im comp pred adeq;

MISSING ARE ALL (999.99);

DEFINE:

pred = (csappa2+csappa3 + csappa5)/3;

adeq= (csappa4+csappa9+csappa10)/3;

ANALYSIS:

ESTIMATOR = MLR

MODEL:

motiv by im* comp pred adeq;

motiv@1;

output: STDYX sampstat tech4 modindices;

# Mplus Syntax for analyses in Step 3

## Four-Factor Correlated CAPL Model

VARIABLE:

NAMES ARE age age_dec pacer plank

camsa step3 step4 self_pa csappa1-csappa5

csappa7-csappa17 ben1-ben9 bar1-bar10

adeq_sum adeq_score pred_sum pred_score

skill_cmprag card_def musc_def pa_is pais_also

Krun Kpushup Kcool Kheart blank_score imprskil

PAQ breq1-breq12 comp1-comp6 comp6_r IM ID IJ

EX compt Ben Bar ratio;

categorical are card_Def musc_def imprskil PAQ;

USEVARIABLES ARE

IM camsa

card_Def musc_def blank_score

imprskil PAQ step44 plankk paccer

comp pred adeq;

MISSING ARE ALL (999.99);

DEFINE:

step44 = step4/1000;

plankk = plank/1000;

Paccer = pacer/1000;

comp = (comp1+comp2+comp5)/3;

Pred = (csappa2+csappa3+csappa5)/3;

Adeq= (csappa4+csappa9+csappa10)/3;

ANALYSIS:

ESTIMATOR = wlsmv;

MODEL:

Physcom by paccer* plankk camsa;

knowledge by card_Def* musc_def blank_score

imprskil PAQ;

step44 with motiv physcom knowledge;

motiv by im* comp adeq pred;

motiv@1;

knowledge@1;

Physcom@1;

output: STDYX sampstat tech4 modindices;

## Hierarchical CAPL Model

VARIABLE:

NAMES ARE age age_dec pacer plank

camsa step3 step4 self_pa csappa1-csappa5

csappa7-csappa17 ben1-ben9 bar1-bar10

adeq_sum adeq_score pred_sum pred_score

skill_cmprag card_def musc_def pa_is pais_also

Krun Kpushup Kcool Kheart blank_score imprskil

PAQ breq1-breq12 comp1-comp6 comp6_r IM ID IJ

EX compt Ben Bar ratio;

categorical are card_Def musc_def imprskil PAQ;

USEVARIABLES ARE

IM camsa

card_Def musc_def blank_score

imprskil PAQ step44 plankk paccer

comp pred adeq;

MISSING ARE ALL (999.99);

DEFINE:

step44 = step4/1000;

plankk = plank/1000;

Paccer = pacer/1000;

comp = (comp1+comp2+comp5)/3;

Pred = (csappa2+csappa3+csappa5)/3;

Adeq= (csappa4+csappa9+csappa10)/3;

ANALYSIS:

ESTIMATOR = WLSMV;

MODEL:

Physcom by paccer* plankk camsa;

knowledge by card_Def* musc_def blank_score

imprskil PAQ;

motiv by im* comp adeq pred;

motiv@1;

knowledge@1;

Physcom@1;

PL by knowledge* physcom step44 motiv;

PL@1;

output: STDYX sampstat tech4 modindices;
