# Supplementary material for: Revising the motivation and confidence domain of the Canadian assessment of physical literacy
Source: BMC Public Health. 2018 Oct 2;18(Suppl 2):1045. doi: 10.1186/s12889-018-5900-0 (PMC6167763; doi:10.1186/s12889-018-5900-0)
Supplement: Supplementary file 6 — Final Motivation and Confidence domain questionnaire. (DOCX 86 kb) [file 12889_2018_5900_MOESM6_ESM.docx]

**What Do You Think About Physical Activity?**

When we ask you about physical activity, we mean when you are moving around, playing or exercising. Physical activity is any activity that makes your heart beat faster or makes you get out of breath some of the time.

**Why are we asking you these questions?**

We want to know what kids like you think about physical activity, sports and exercise.

**Please remember:**


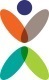
 There are no right or wrong answers! We only want to know what you think.


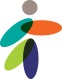
 If you do not know an answer, please write your best guess.


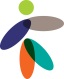
 There is no time limit, so please take all the time you need.

**What’s Most Like Me?**

For the first questions you have to read two sentences and then circle the sentence you think is **MORE LIKE YOU**.

Try the following **SAMPLE QUESTION**:

| **Some kids have one nose on their face!** | **BUT** | **Other kids have three noses on their face!** |
| --- | --- | --- |

That shouldn’t be too hard for you to decide! Once you have circled the sentence that is more like you, then you have to decide if it is **REALLY TRUE** for you or **SORT OF TRUE** for you.

Here is another sample question for you to try. Remember, to answer the question you need to do two things:

1. **First circle the sentence that is more like you.**
2. **Then put a check in the correct box if it is really true or only sort of true for you**.

THERE ARE NO RIGHT OR WRONG ANSWERS, JUST TELL US WHAT YOU THINK IS
**MOST LIKE YOU**.

**SAMPLE QUESTION #2**:

| **Some kids like to play with computers** | | **BUT** | **Other kids don’t like playing with computers** | |
| --- | --- | --- | --- | --- |
| **□ REALLY TRUE for me** | **□ SORT OF TRUE for me** |  | **□ REALLY TRUE for me** | **□ SORT OF TRUE for me** |

Now you are ready to start filling in this form**. Remember, in each box you need to circle what is most like you and then check a box for “really” or “sort of” true**. Take your time and do the whole form carefully. If you have any questions, just ask! If you think you are ready you can start now.

BE SURE TO FILL IN EACH PAGE!

**What’s Most Like Me?**

| **Some kids don’t like playing active games** | | **BUT** | **Other kids really like playing active games** | |
| --- | --- | --- | --- | --- |
| **□ REALLY TRUE  for me** | **□ SORT OF TRUE  for me** |  | **□ REALLY TRUE  for me** | **□ SORT OF TRUE  for me** |

| **Some kids are good at active games** | | **BUT** | **Other kids find active games hard to play** | |
| --- | --- | --- | --- | --- |
| **□ REALLY TRUE   for me** | **□ SORT OF TRUE   for me** |  | **□ REALLY TRUE   for me** | **□ SORT OF TRUE   for me** |

| **Some kids don’t have much fun playing sports** | | **BUT** | **Other kids have a good time playing sports** | |
| --- | --- | --- | --- | --- |
| **□ REALLY TRUE   for me** | **□ SORT OF TRUE   for me** |  | **□ REALLY TRUE   for me** | **□ SORT OF TRUE   for me** |

| **Some kids do well in most sports** | | **BUT** | **Other kids feel they aren’t good at sports** | |
| --- | --- | --- | --- | --- |
| **□ REALLY TRUE   for me** | **□ SORT OF TRUE   for me** |  | **□ REALLY TRUE   for me** | **□ SORT OF TRUE   for me** |

| **Some kids don’t like playing sports** | | **BUT** | **Other kids really enjoy playing sports** | |
| --- | --- | --- | --- | --- |
| **□ REALLY TRUE   for me** | **□ SORT OF TRUE   for me** |  | **□ REALLY TRUE   for me** | **□ SORT OF TRUE   for me** |

| **Some kids learn to play active games easily** | | **BUT** | **Other kids find it hard learning to play active games** | |
| --- | --- | --- | --- | --- |
| **□ REALLY TRUE   for me** | **□ SORT OF TRUE   for me** |  | **□ REALLY TRUE   for me** | **□ SORT OF TRUE   for me** |

**Thank you for telling us which kids are most like you!**

We have just a few more questions about physical activity.

Please turn to the next page.

**Why are you active?**

Boys and girls can be **active** by doing all sorts of things:

- Exercise (walking, keeping fit, or gym class)
- Playing outside or doing active things (like playing in the park)
- Sports (like soccer, tennis, hockey, dance or swimming)

Below are some reasons why you might be active.

Please read each sentence and tell us how true it is for you.

| **I am active because...** | | | | | |
| --- | --- | --- | --- | --- | --- |
|  | Not true for me | Not really true for me | Sometimes true for me | Often true for me | Very true for me |
| being active is fun | € | € | € | € | € |
| I enjoy being active | € | € | € | € | € |
| I like being active | € | € | € | € | € |

**How do you feel about being active?**

The next section has some sentences describing how girls and boys feel about BEING ACTIVE and DOING ACTIVE THINGS (like active games, playing outside and doing sports).

Please read each sentence and tell us how much each sentence is like you.

|  | Not like me at all | Not really like me | Sometimes like me | Quite a lot like me | Really like me |
| --- | --- | --- | --- | --- | --- |
| When it comes to playing active games, I think I am pretty good. | € | € | € | € | € |
| I think I do well compared to other children | € | € | € | € | € |
| When it comes to being active, I have good skills. | € | € | € | € | € |
