# Supplementary material for: Revising the motivation and confidence domain of the Canadian assessment of physical literacy
Source: BMC Public Health. 2018 Oct 2;18(Suppl 2):1045. doi: 10.1186/s12889-018-5900-0 (PMC6167763; doi:10.1186/s12889-018-5900-0)
Supplement: Supplementary file 7 — Exploratory models. (DOCX 16 kb) [file 12889_2018_5900_MOESM7_ESM.docx]

**Additional File 7**

**Table S1**

Model Fit statistics for additional exploratory models of motivation and confidence

|  |  | Chi-Square | df | P | CFI | RMSEA | RMSEA CI |
| --- | --- | --- | --- | --- | --- | --- | --- |
| Option 5 |  | 13.21 | 5 | .02 | .927 | .090 | .032, .150 |
| Option 6 |  | 1.198 | 2 | .55 | 1.0 | 0.0 | 0.0, .0119 |
| Option 7 |  | 6.713 | 5 | .24 | .989 | .041 | .00, .11 |

**Note.** Model 5 = composite scores of intrinsic motivation, skills compared to peers, shortened adequacy and shortened predilection, and a behaviour subscale. Model 6 = composite scores of intrinsic, identified, skills compared to peers, and perceived benefits. Model 7 = composite scores of intrinsic motivation, identified motivation, perceived competence satisfaction shortened adequacy and shortened predilection). df = degrees of freedom, CFI = comparative fit index, RMSEA = Root Mean Square Error of Approximation, CI = confidence interval.
